# Local-strain-induced CO_2_ adsorption geometries and electrochemical reduction pathway shift

**DOI:** 10.1093/nsr/nwae191

**Published:** 2024-06-07

**Authors:** Chuhao Liu, Yifan Bu, Yifei Xu, Azhar Mahmood, Jisheng Xie, Yifan Fu, Shiyun Li, Cheng Peng, Yue Wu, Xiao Liang, Ruilong Zong, Wan-Lu Li, Jihan Zhou, Bingjun Xu, Li Niu, Mufan Li

**Affiliations:** College of Chemistry and Molecular Engineering, Peking University, Beijing 100871, China; College of Chemistry and Molecular Engineering, Peking University, Beijing 100871, China; College of Chemistry and Molecular Engineering, Peking University, Beijing 100871, China; School of Chemistry and Chemical Engineering, Guangzhou University, Guangzhou 510006, China; College of Chemistry and Molecular Engineering, Peking University, Beijing 100871, China; College of Chemistry and Molecular Engineering, Peking University, Beijing 100871, China; College of Chemistry and Molecular Engineering, Peking University, Beijing 100871, China; College of Chemistry and Molecular Engineering, Peking University, Beijing 100871, China; College of Materials Science and Engineering, Fuzhou University, Fuzhou 350108, China; Department of Chemistry, Tsinghua University, Beijing 100084, China; Department of Chemistry, Tsinghua University, Beijing 100084, China; Department of NanoEngineering, University of California, San Diego, La Jolla 92093, USA; College of Chemistry and Molecular Engineering, Peking University, Beijing 100871, China; College of Chemistry and Molecular Engineering, Peking University, Beijing 100871, China; School of Chemistry and Chemical Engineering, Guangzhou University, Guangzhou 510006, China; College of Chemistry and Molecular Engineering, Peking University, Beijing 100871, China

**Keywords:** local strain, CO_2_ electroreduction, pathway shift, PdCu alloys

## Abstract

Unravelling the influence of strain and geometric effects on the electrochemical reduction of carbon dioxide (CO_2_RR) on Cu-based (or Pd-based) alloys remains challenging due to complex local microenvironment variables. Herein, we employ two PdCu alloys (nanoparticles and nanodendrites) to demonstrate how CO_2_RR selectivity can shift from CO to HCOO^−^. Despite sharing consistent phases, exposed crystal facets, and overall oxidative states, these alloys exhibit different local strain profiles due to their distinct geometries. By integrating experimental data, *in-situ* spectroscopy, and density functional theory calculations, we revealed that CO_2_ prefers adsorption on tensile-strained areas with carbon-side geometry, following a *COOH-to-CO pathway. Conversely, on some compressive-strained regions induced by the dendrite-like morphology, CO_2_ adopts an oxygen-side geometry, favoring an *OCHO-to-HCOO pathway due to the downshift of the *d*-band center. Notably, our findings elucidate a dominant *OCHO-to-HCOO^−^ pathway in catalysts when featuring both adsorption geometries. This research provides a comprehensive model for local environment of bimetallic alloys, and establishes a clear relationship between the CO_2_RR pathway shift and variation in local strain environments of PdCu alloys.

## Teaser

Complex nano-geometries are always accompanied by different local-environments. This work reveales that geometry variations dominate the local strain change, resulting in a shift in the electrochemical reduction reaction path of CO2.

## INTRODUCTION

Activation and electrochemical reduction of carbon dioxide (CO_2_RR) into high-value chemical feedstocks, with renewable energy, emerges as a plausible approach to simultaneously address the pressing issues of climate change and energy deficiency [1,2]. Generally, CO_2_ molecules interact with catalytic surfaces by adhering to two distinct geometries, defining the forthcoming reaction pathways. The two adsorptive configurations of CO_2_ can be activated into two principal geometries—*COOH and *OCHO intermediates. The first pathway involves binding a carbon atom to the catalytic surface, while the other pathway primarily engages the oxygen atom [3–8]. In the case of the former, it converts CO_2_ into *CO intermediate, potentially generating C_2+_ hydrocarbons [9,10] or oxygenates [11] via a C–C coupling and hydrogenation process. Conversely, the latter pathway instigates the formation of formic acid/formate [12], formaldehyde [13], and other products. Furthermore, cascading these products with bio-catalysis [2,14] or abio-catalysis [15] can even upcycle CO_2_ into carbohydrates.

Copper is still the most functional electrocatalyst for deeply catalyzing CO_2_RR [16]. Nevertheless, a fraction of high-activity undercoordinated sites of pure metallic Cu tend to decay under electrochemical conditions [17,18]. Alloying is extensively employed to tune the catalytic activity and corrosion resistance for CO_2_RR [19–23]. However, disentangling strain effects from the accompanied geometric effects of alloying is still a challenge [8,24]. Given that most characterization techniques can only obtain overall information about the material [25], local environment changes are difficult to spot in many cases. For example, irrespective of their state of order, disorder, phase-separation, or atomic arrangement, PdCu nanoparticles (PdCu-NP) show a proclivity towards the *COOH-mediated pathway [20,26–28]. However, PdCu nanodendrites (PdCu-ND) with even the same elemental composition and electronic structures have demonstrated substantial activity towards the *OCHO-to-formate pathway [21,29] (Table S1). Therefore, we need to further explore the potential impact of local environments’ change on the performance of CO_2_RR [30,31].

In our investigation, we meticulously synthesized PdCu-NP and PdCu-ND via the wet-chemical method [32,33], ensuring a consistent oxidative state, phase, and exposed crystal facets across the samples. The PdCu-NP exhibited effective CO_2_-to-CO activity, while the PdCu-ND transitioned to commendable CO_2_-to-formate performance within the same potential range. By careful characterization, we found that the morphology difference is accompanied by a distinct local-environment configuration (LEC). One LEC occurs on PdCu-NP and the branch part of PdCu-ND and owns a tensile strain characteristic, while the other LEC has distinct compressive strain on the joint part of PdCu-ND. By integrating *in situ* electrochemical attenuated total reflectance surface enhanced infrared absorption spectroscopy (ATR-SEIRAS) spectra with density functional theory (DFT) calculations, we discerned that CO_2_ tends to be adsorbed on the former tensile LEC by a carbon-side geometry and activated through a *COOH-to-CO pathway, while on the latter compressive LEC, it tends to be adsorbed by an oxygen-side geometry and activated through an *OCHO-to-HCOO^−^ pathway. Intriguingly, our findings disclose a preferential *OCHO-to-HCOO^−^ pathway when both routes are present on one catalyst. This work provided a new paradigm for the study of the local environment, and unraveled the relationship between the CO_2_RR pathway shift and local strain variation on bimetallic alloys.

## RESULTS AND DISCUSSION

### Synthesis and structural characterization of PdCu-NP and PdCu-ND

**Figure 1.**
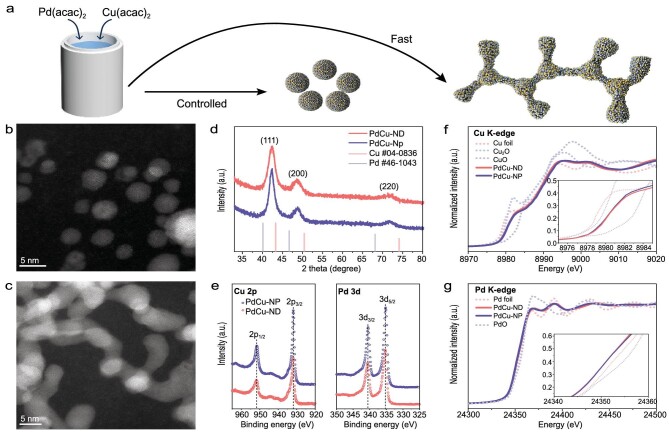
Synthesis and structural characterization. (a) Scheme of the synthesis process of PdCu-NP and PdCu-ND. (b, c) HR-HAADF-STEM images of PdCu-NP and PdCu-ND and corresponding (d) XRD and (e) XPS. XANES at (f) Cu K-edge and (g) Pd K-edge.

We meticulously prepared PdCu-NP and PdCu-ND by reducing the same proportion of metal precursors, palladium (II) acetylacetonate (Pd(acac)_2_) and copper(II) acetylacetonate (Cu(acac)_2_), under the same experimental conditions [33] (see Methods for details). The only controlled step is that we employed a different amount of additional reducing agent (ascorbic acid, AA) to control the reduction rate of metal precursors and the resultant morphology changes from particles to dendrites (Fig. 1a and Fig. S1).

The high-angle annular dark-field scanning transmission electron microscopy (HAADF-STEM) and high-resolution TEM (HRTEM) images show that the morphology of PdCu-NP is sphere-like nanoparticles (∼3–5 nm) (Fig. 1b and Fig. S2) while PdCu-ND exhibits a dendrite-like morphology (Fig. 1c). The HRTEM images and diffraction patterns (Figs S3–S5) suggest that Pd and Cu formed an alloy face centered cubic (fcc) structure as indicated by a *d*-spacing of 2.20 ± 0.01 Å corresponding to a (111) interplanar distance in PdCu-NP and PdCu-ND. The X-ray diffraction (XRD) peaks (Fig. 1d) of the two alloys exhibited the same diffraction peaks at 42.05°, 48.55° and 71.37°, which are assigned to the (111), (200) and (220) planes of the fcc structure [20], which agrees with the HRTEM images and diffraction patterns.

Furthermore, we employed X-ray photoelectron spectroscopy (XPS) and X-ray absorption spectroscopy (XAS) to study the average oxidative states and overall coordination states across the samples. The XPS spectra (Fig. 1e and Fig. S6) show that, in PdCu-NP and PdCu-ND, Pd and Cu exhibit very similar chemical valence states possibly due to that ligands (such as KI) stabilize metal valence states. The normalized X-ray absorption near edge structure spectra (XANES) of the Cu K-edge (Fig. 1f) show that the near-edge lines for PuCu-NP and PdCu-ND are overlapped and close to Cu_2_O. The XANES of the Pd K-edge (Fig. 1g) also show that PdCu-NP and PdCu-ND alloys exhibit equal near edge features and white-line intensity, which reveals that the overall Pd oxidative states are the same. These observations agree with the XPS results. Generally, the synthesized PdCu-NP and PdCu-ND, own consistent phase, exposed crystal facets, and oxidative state across the samples.

### Local environment characterization and analysis

**Figure 2.**
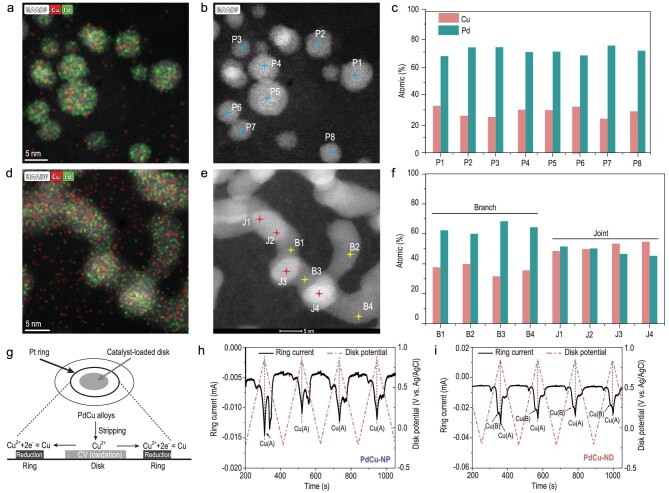
Local environment characterization and analysis. (a, d) Overlapped EDS elemental mapping with (b, e) HR-HAADF-STEM images and corresponding (c, f) elemental distribution of PtCu-NP and PdCu-ND, respectively. (g) Scheme of Cu stripping and collection and testing for (h) PdCu-NP and (i) PdCu-ND in 0.05 M H_2_SO_4_.

In order to portray the local environment of PdCu-NP and PdCu-ND, we performed detailed energy dispersive spectroscopy (EDS). The overlapped EDS elemental mapping with HR-HAADF-STEM images (Fig. 2a–f and Fig. S7) shows that Pd and Cu are evenly distributed throughout the catalysts. We also carried out a detailed EDS spot scan and found that the Cu/Pd ratio is close to 25/75 on particles (Fig. 2c and Figs S8, S9). Specially, on the PdCu-ND, the composition arrangement on branch parts (denoted as B, the average Cu/Pd ratio is 36/64) are similar to that of PdCu-NPs, while Cu is enriched on the joint part (denoted as J, the average Cu/Pd ratio is 52/48) (Fig. 2f and Fig. S10). Therefore, in the case of element distribution, the Pd-rich PdCu-NP are similar to the branch parts of PdCu-ND while the joint parts of PdCu-ND are Cu-rich. In other words, the geometry variations are accompanied by local component differences.

In addition, we developed an electrochemical Cu stripping and collection method to portray the local environment of PdCu-ND and PdCu-NP. As shown in the scheme (Fig. 2g), PdCu alloys were loaded on the disk which applied oxidative potential (from −0.214 V to 0.85 V versus Ag/AgCl); meanwhile, the Pt-ring was biased at a constant reductive potential (−0.17 V versus Ag/AgCl) in order to collect oxidized Cu ions. During the forward scanning process of cyclic voltammetry (CV) on the disk, mainly only one kind of Cu (denoted as Cu(A)) was detected at 0.85 V versus Ag/AgCl in PdCu-NP (Fig. 2h and Fig. S11). For PdCu-ND, in addition to Cu(A), there was another Cu (denoted as Cu(B)) detected at 0.70 V versus Ag/AgCl (Fig. 2i and Fig. S11). The electrochemical Cu stripping and collection further proved that, for Cu atoms, the local environment of PdCu-NP and branch part of PdCu-ND are similar, while the joint part of PdCu-ND is different.

**Figure 3.**
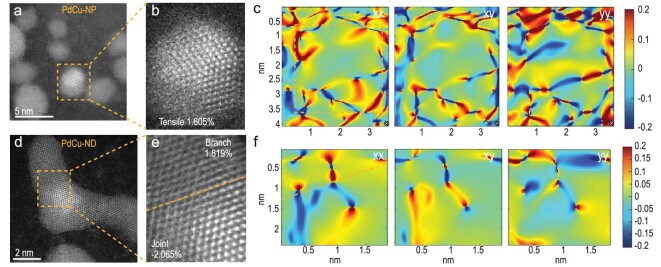
Local environment characterization and analysis. (a–c) PdCu-NP's and (d–f) PdCu-ND's AC-HADDF-STEM images and corresponding strain maps at the tensor of xx, xy and yy, respectively.

Furthermore, we employed geometric phase analysis (GPA) [34] to analyze the strain of PdCu-NP and PdCu-ND. As for PdCu-NP, maps of in-plain strain of GPA show that the nanoparticles are mostly dominated by tensile strain (Fig. 3a–c), and the statistical average strain (ε_xx_) of nanoparticles is 1.605%. When it comes to PdCu-ND, the branch part exhibits 1.819% tensile strain as does PdCu-NP; however, the joint part of PdCu-ND is mostly dominated by the compressive strain of −2.06% (Fig. 3d–f). These local strain variations are also found along with the gradual morphology change from particles to dendrites (Fig. S12). In addition, we supplemented the wavelet transformed (WT) EXAFS spectra (Fig. S13) and found that the centers of [χ(k), χ(R)] intensity of PdCu-ND are lower than that of PdCu-NP, which indicates the relatively compressive character of PdCu-ND. The results are consistent with the overall strain analysis.

### Electrochemical performance and structure-activity relationship

**Figure 4.**
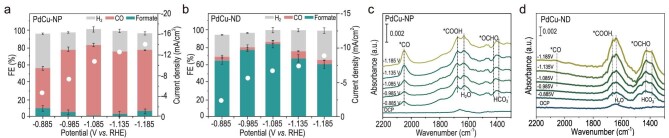
Performance and structure-activity relationship. (a, b) FE and current density of CO_2_RR performance for PdCu-NP and PdCu-ND and (c, d) corresponding *in situ* ATR-SEIRAS spectra.

When it comes to the performance of electrochemical CO_2_RR, we conducted tests using a CO_2_-saturated 0.1 M KHCO_3_ solution. As shown in the linear sweep voltammetry (LSV) curves (Fig. S14), PdCu-NP displayed a higher current density than PdCu-ND over the entire potential range, which may derive from higher surface areas (Fig. S15). When it comes to CO_2_ reduction products, the synthesized PdCu alloys have good CO_2_ fixation ability (Fig. S16). The PdCu-NP exhibited effective CO_2_-to-CO activity (>80% faradaic efficiency (FE_CO_), at −1.085 V versus reversible hydrogen electrode (RHE)) (Fig. 4a). For PdCu-Mid, we can find that both a certain amount of formate and CO were produced over the whole potential window (Fig. S17), while the PdCu-ND transitioned to commendable CO_2_-to-formate performance (>80% ${\mathrm{F}}{{{\mathrm{E}}}_{{\mathrm{HCO}}{{{\mathrm{O}}}^ - }}}$, at −1.085 V versus RHE) within the same potential range (Fig. 4b and Fig. S18). After a 24-h reaction, both catalysts showed relatively good morphology stability and selectivity (Figs S19–S21). These performances are consistent with other independently reported PdCu nanoparticles [20,26–28] or PdCu nanodendrites [21,29].

Besides, to further figure out the roles of Pd and Cu sites, the CO_2_RR performance of pure Pd (Fig. S22a) was tested under the same conditions, and results showed that H_2_, CO, and formic acid can be detected in the product, but as the voltage increases, H_2_ begins to dominate (Fig. S22b). To assess this further, we employed CO stripping in order to discover whether Pd sites of PdCu alloys can act with CO or not. The CO stripping results (Fig. S23) showed that there is no obvious CO oxidation on PdCu alloys, which should be located at ∼0.88 V versus RHE. Therefore, we suppose that the Cu site is likely to play a role in adsorption and activation of CO_2_, while the Pd site is better at providing protons for CO_2_ hydrogenation.

To further distinguish the component or the strain that determines the CO_2_RR performance difference, we synthesized Pd_1_Cu_1_-NP (see Supplementary method for details) as a control catalyst (Figs S24 and S25). Though Pd_1_Cu_1_-NP has a similar Pd and Cu amount with the joint part of PdCu-ND, the GPA results (Fig. S26) showed that Pd_1_Cu_1_-NP has a tensile strain (1.63%). The CO_2_RR performance of Pd_1_Cu_1_-NP (Fig. S27) showed that the main product is still CO, the same as tensile PdCu-NP's (Table 1). Therefore, we concluded that the compressive strain of the joint part of PdCu-ND probably derived from the morphology effect but not component difference, and the tensile local environment of PdCu alloys are conducive to CO_2_-to-CO pathways.

**Table 1.** The strains and components of different PdCu alloys.

Catalyst	Cu/Pd ratio	Strain	Main product
PdCu-NP	25.25/74.75	Tensile	CO
Pd_1_Cu_1_-NP	47.64/52.36	Tensile	CO
PdCu-ND (Branch)	36.20/63.80	Tensile	Formate
PdCu-ND (Joint)	51.56/48.44	Compressive	

We then employed *in situ* ATR-SEIRAS for further investigation (Fig. S28). In CO_2_-saturated 0.1 M NaHCO_3_ solution, we used the infrared signal intensity measured at open-circuit potential (OCP) as the background. In Fig. 4c, on scanning the applied potential from −0.885 to −1.185 V versus RHE over the PdCu-NP catalyst, a band at 2050 cm^−1^ attributable to CO (derived from *COOH intermediates [4,5,35]) adsorbed atop sites of the Cu [12,18] was observed, which is in agreement with recently reported tensile Cu [36]. A combination peak at 1670–1675 cm^−1^ can be assigned to the C=O asymmetric stretch of *COOH intermediate [35] and the inverse water peak [37] (H–O–H bend, at 1630–1640 cm^−1^). The band located at 1670–1675 cm^−1^ can be attributed to the vibration band of HCO_3_^−^ [38]. The peaks centered around 1420–1430 cm^−1^ can be assigned to *OCHO species [39,40], which is the key intermediate for formate formation. On PdCu-ND, because it owns both compressive joint and tensile branch, we can observe both *OCHO and *COOH intermediate. The relative intensity of *OCHO on PdCu-ND is much more obvious because HCOO^−^ dominates the products. To sum up, *in situ* ATR-SEIRAS results of the two catalysts are highly consistent with the corresponding CO_2_RR performance, which confirmed the CO_2_-*COOH-CO pathway on tensile areas, while the CO_2_-*OCHO-formate pathway occurs when a compressive local environment coexists in PdCu alloys.

### Theoretical study

**Figure 5.**
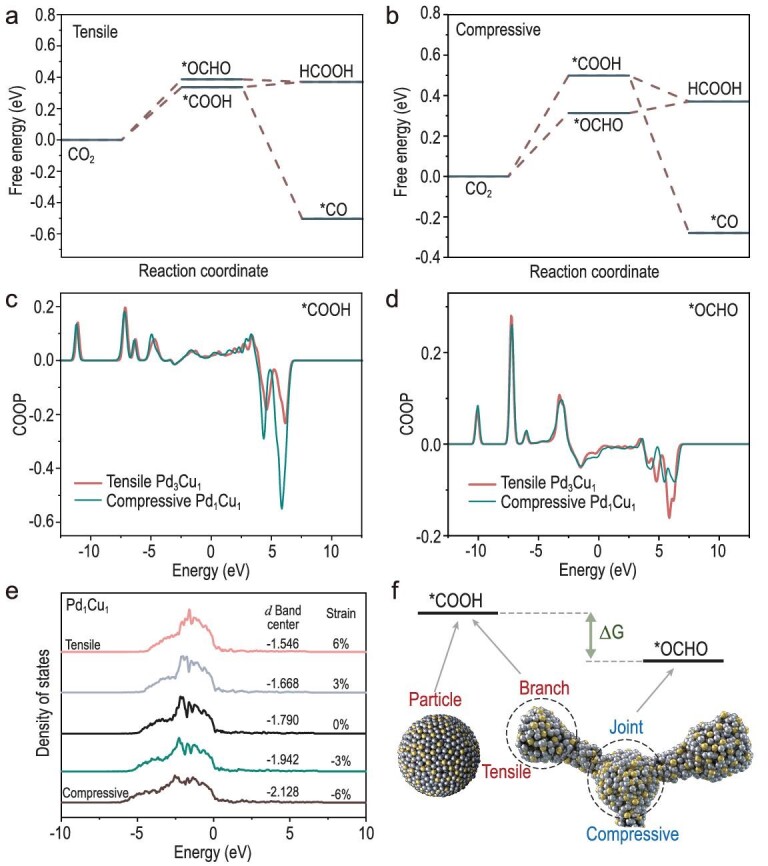
Theoretical study. The DFT calculation on (a) the tensile Pd_3_Cu_1_, and (b) compressive Pd_1_Cu_1_. The COOP of (c) *COOH and (d) *OCHO on tensile Pd_3_Cu_1_ and compressive Pd_1_Cu_1_. (e) The *d* band centers change of Pd_1_Cu_1_ along with different strains. (f) Scheme of the mechanism.

Furthermore, we constructed structural models based on the metal composition of PdCu alloys and conducted DFT calculations to explore the structure–activity relationship. According to the strain and selectivity characteristic, we constructed tensile Pd_3_Cu_1_ in order to represent the structure of PdCu-NP and the branch part of PdCu-ND, and compressive Pd_1_Cu_1_ to represent the joint part of PdCu-ND (Fig. S29). Fig. 5a shows that, on tensile Pd_3_Cu_1_ surface, the free energy of *COOH is lower than that of *OCHO by 0.05 eV, which is consistent with the fact that the PdCu-NP exhibited effective CO_2_-*COOH-CO activity. When it comes to compressive Pd_1_Cu_1_ surface (Fig. 5b), the free energy gap between *OCHO and *COOH became larger, with the free energy of *OCHO lower than that of *COOH by 0.2 eV. Besides, the free energy of *OCHO on the joint part is 0.313 eV which is also lower than 0.337 eV of *COOH on the branch part. It explained the reason why PdCu-ND exhibited the CO_2_-*OCHO-formate pathway mainly in a certain portion of the tensile branch part. We further simulated a larger strain and found the same trend (Fig. S30).

Besides, crystal orbital overlap population [41] (COOP) analysis also evidenced that *COOH (Fig. 5c) tends to form on tensile Pd_3_Cu_1_ while *OCHO (Fig. 5d) tends to form on compressive Pd_1_Cu_1_. Integrated COOP (ICOOP) of different intermediates (Table S2) follows: (1) *COOH: tensile Pd_3_Cu_1_ > compressive Pd_1_Cu_1_; (2) *OCHO: tensile Pd_3_Cu_1_ < compressive Pd_1_Cu_1_, further supporting our conclusions. The reason is most likely the greater downshift of *d*-band center of compressive Pd_1_Cu_1_ which leads to a higher ratio of *OCHO to *COOH coverage (Fig. 5e and Fig. S31) [42]. In other words, the DFT results showed that the CO_2_-*COOH-CO pathway occurred on tensile PdCu-NP, while the CO_2_-*OCHO-formate pathway was dominant on the compressive joint part of PdCu-ND and the CO_2_-*COOH-CO pathway was suppressed on the tensile branch part (Fig. 5f).

## CONCLUSION

In summary, we synthesized PdCu-NP and PdCu-ND with a consistent oxidative state, phase, and exposed crystal facets as model catalysts to regulate CO_2_RR reaction pathways. By careful characterization, we found that the local compressive strain in the joint part of PdCu-ND is generated by morphology effects, and the tensile strain is conductive to the CO_2_-*COOH-CO pathway while the CO_2_-*OCHO-formate pathway occurs preferentially when compressive strain co-exists because of the lower free energy of the formation of *OCHO than that of *COOH. Therefore, the CO_2_RR selectivity shifts from CO to formate as the CO_2_-*OCHO-formate pathway is dominant on the joint part of PdCu-ND, though on the branch part of PdCu-ND has the chance to conduct the CO_2_-*COOH-CO pathway. This work provided a new paradigm for the study of the local environment of bimetallic alloys, and unraveled the relationship between the CO_2_RR pathway shift and local strain variation of PdCu alloys.

## METHODS

### Chemicals and materials

Palladium acetylacetonate, copper acetylacetonate (Cu(acac)_2_, 99.9%), poly(vinylpyrrolidone) (PVP, MW = 10 000), potassium iodide (KI, 99.99%), *L*-ascorbic acid (99.9%), tris(hydroxymethyl) aminomethane (99.8%), formaldehyde solution (HCHO, 37%) and formamide (99.5%) were purchased from Sigma-Aldrich. Acetone (99.9%) and ethanol (99.9%) were purchased from Sinopharm Chemical Reagent. All the materials were used as received without further purification for the synthesis of PdCu alloys.

### Synthesis of PdCu nanoparticles

In a typical synthesis of PdCu nanoparticles, a mixture of 100 mg of tris and 400 mg of PVP was dissolved in 3 mL of HCHO solution and transferred to Teflon-lined stainless-steel autoclave which was heated at 200°C for 3 h. A gel-like material was obtained after washing and centrifugation in acetone. A homogeneous solution of 0.02 mmol of Pd(acac)_2_, 0.02 mmol of Cu(acac)_2_ and 80 mg/g of KI and ascorbic acid (5 mg) was prepared in 4 mL of formamide solvent and poured into a 12-mL Teflon-lined stainless-steel autoclave along with the gel-like material which was prepared earlier; the autoclave was then kept in the oven at 150°C for 3 h. The final product was obtained after washing with ethanol and acetone.

### Synthesis of PdCu nanodendrites

Synthesis of PdCu nanodendrites was carried out under the same experimental conditions as nanopartcles, except that the ligand (i.e. KI) and reducing agent (i.e. ascorbic acid) was added along with Pd(acac)_2_, Cu(acac)_2_, and the amount of ascorbic acid used was increased to 80 mg.

## Supplementary Material

nwae191_Supplemental_File
