# Supplementary material for: Local-strain-induced CO2 adsorption geometries and electrochemical reduction pathway shift
Source: Natl Sci Rev. 2024 Jun 7;11(12):nwae191. doi: 10.1093/nsr/nwae191 (PMC11562823; doi:10.1093/nsr/nwae191)
Supplement: nwae191_Supplemental_File [file nwae191_supplemental_file.docx]

**Supporting Information for**

**Local strain induced CO_2_ adsorption geometries and electrochemical reduction pathways shift**

Chuhao Liu^1†^, Yifan Bu^1†^, Yifei Xu^1†^, Azhar Mahmood^2*^, Jisheng Xie^1^, Yifan Fu^1^, Shiyun Li^1^, Peng Cheng^1^, Yue Wu^3^, Xiao Liang^4^, Ruilong Zong^4^, Wan-Lu Li^5^, Jihan Zhou^1^, Bingjun Xu^1^, Li Niu^2*^ and Mufan Li^1^*

^1^ College of Chemistry and Molecular Engineering, Peking University, Beijing 100871, China.

^2^ School of Chemistry and Chemical Engineering, Guangzhou University, Guangzhou 510006, China.

^3^ College of Materials Science and Engineering, Fuzhou University, Fuzhou 350108, China.

^4^ Department of Chemistry, Tsinghua University, Beijing 100084, China.

^5^ Department of NanoEngineering, University of California San Diego, La Jolla, CA 92093, USA.

^†^ These authors contributed equally to this work.

^∗^ Corresponding authors. E-mails: azhar@gzhu.edu.cn; lniu@gzhu.edu.cn; mufanli@pku.edu.cn

**METHODS**

**Synthesis of Pd_1_Cu_1_-NP.** The synthesis method of Pd_1_Cu_1_-NP is as same as PdCu-NP but adding three times as much as Cu precusors.

**Synthesis of pure Pd-NP.** The synthesis method of pure Pd is as same as that of PdCu-NP but without adding Cu precusors.

**CHARACTERIZATION**

The X-ray diffraction (XRD) were performed using a PW3040/60 PANalytical with Cu Kα as an X-ray source. The transmission electron microscopy (TEM), high-angle annular dark-field (HAADF) STEM images and the corresponding energy dispersive spectrometer (EDS) were collected on a JEOL JEM-2100F 200kV field emission transmission electron microscope equipped with an OXFORD EDS. The X-ray photoelectron spectroscopy (XPS) were collected on ESCALAB 250Xi with Al Kα radiation (1486 eV) as a probe. The X-ray Absorption Near Edge Structure (XANES) and Extended X-ray Absorption Fine Structure (EXAFS) data were collected at Beamline 11B at Shanghai Synchrotron Radiation Facility (SSRF) and 1W1B station in Beijing Synchrotron Radiation Facility (BSRF) and the RapidXAFS 2M (Anhui Absorption Spectroscopy Analysis Instrument Co., Ltd.).

**STRAIN DETERMINATION**

The strain values of derived PdCu samples were calculated using the equation:

Strain (%) = *(d-d_0_)/d_0_* * 100%

where *d* is the d-spacing of a given reflection determined from the HAADF-STEM and *d_0_* is the corresponding average d-spacing. And software CrysTBOX is employed for strain analysis.

**CATALYTIC EVALUATIONS**

The electrochemical CO_2_RR performance was evaluated in a three-electrode setup using a two-compartment electrochemical cell with CO_2_-saturated 0.1 M KHCO_3_ solution (pH = 6.8). The gas products were quantified by gas chromatography. The liquid phase products are quantified by 500M HZ NMR.

**THE TOF VALUES OF CO_2_RR PERFORMANCE.**

The turnover frequency (TOF) was evaluated by the following standard equation: TOF = (J × A) / (2 × F × n). Where J (A/cm^2^) is the partial current for certain product; A is the geometric surface area of the electrode; F stands for the Faraday constant, 96485 C/mol; n (mol) is molar amount of active site (Cu) loaded on the carbon paper electrode.

**CO STRIPPING** **EVALUATIONS**

The CO strriping was carried out in 0.1M KOH solution with casting PdCu alloys onto RDE as working electrode, graphite rod as counterelectrodes and Hg/HgO as reference.

***in situ* ATR-SEIRAS EXPERIMENTS.**

First, we coated the PdCu alloys on the surface enhanced Au film. In CO_2_-saturated 0.1 M NaHCO_3_ solution, we used the infrared signal intensity measured at open-circuit potential (OCP) as the background. Then we measured the infrared signal intensity at different potentials from -0.885V to -1.185 V. In the *in situ* ATR-SEIRAS spectra obtained under different potentials, a negative peak indicates that a certain substance or functional group is consumed or has disappeared, and a positive peak indicates that a certain substance or functional group is produced or has increased.

**COMPUTATIONAL DETAILS**

We used the Vienna Ab Initio Simulation Package (VASP)[1-3] to perform the density functional calculations. Projector augmented wave with the generalized gradient approximation of Perdew–Burke–Ernzerhof (PAW-PBE) potentials were used. D3 correction was used to describe London dispersion [4, 5]. A (2×2) surface unit cell of metal (111) with correspondence ratio and strain was modeled with 4 layers to simulate the experiment situation. A vacuum layer of 20 Å was built. Brillouin zone was sampled using a 3 × 3 × 1 Monkhorst–Pack grid[6]. Cutoff energy was chosen to be 450 eV. The force convergence criterion was chosen to be 0.02 eV Å^−1^. We calculated Gibbs free by the equation

$$\Delta G = \Delta E + \Delta ZPE-T \times\Delta S$$

where $\Delta E$ is the reaction energy, $\Delta ZPE$ is the changes in zero-point energies, and $\Delta S$ is the entropy during the reaction. After optimization, the strain for Pd_1_Cu_1_ and Pd_3_Cu_1_ were -0.4% and 5%, respectively.

Supplementary Figure

**
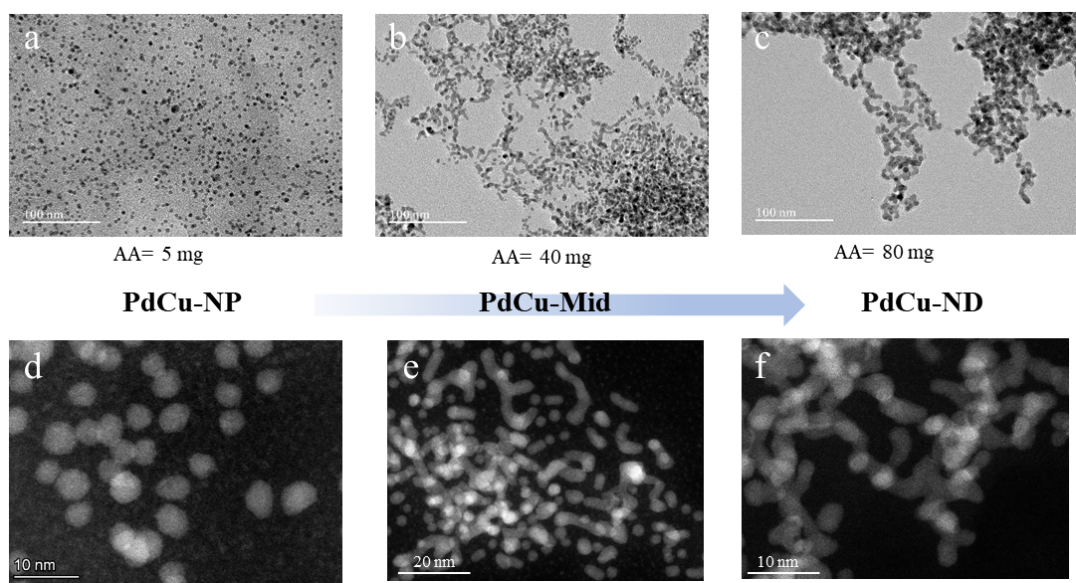
**

**Figure S1.** (**a-c**) TEM images and (**d-f**) HR-HAADF-STEM images of PdCu alloys, the morphology changes form particles to dendrites gradually.


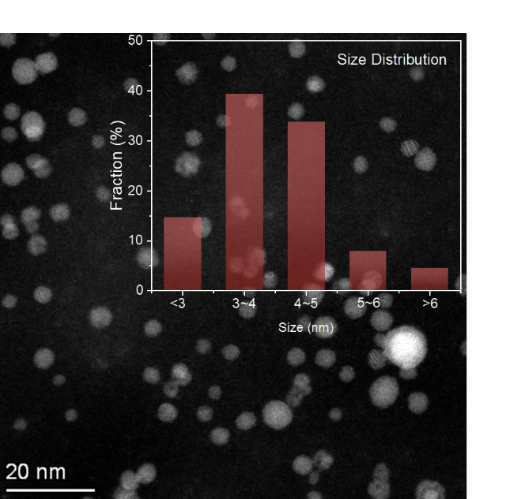


**Figure S2.** The size distribution of PdCu-NP. The size of partucles is mainly distributed in 3~5 nm (a total of 73.03%).


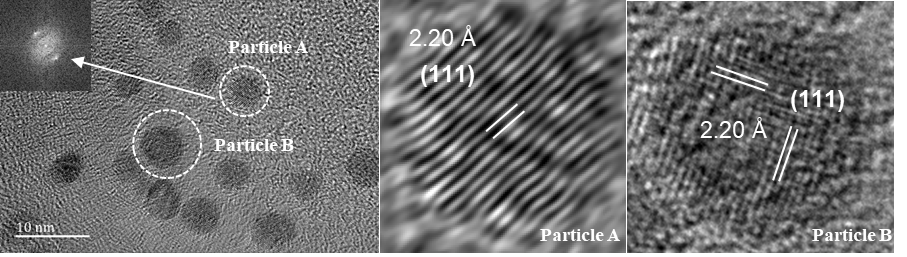


**Figure S3.** HRTEM images of PdCu-NP (the inset shows the corresponding FFT image) and corresponding representative particles and exposed crystal facets.


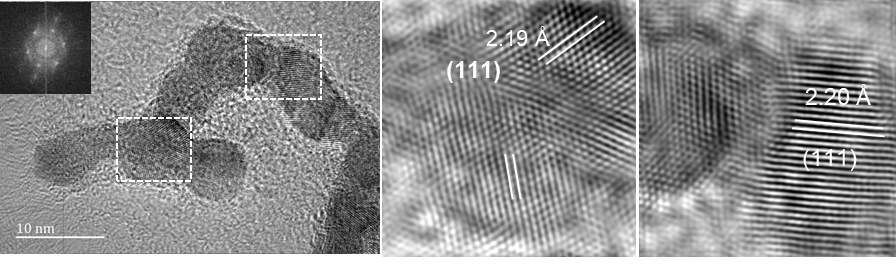


**Figure S4.** HRTEM images of PdCu-ND (the inset shows the corresponding FFT image) and corresponding representative particles and exposed crystal facets.


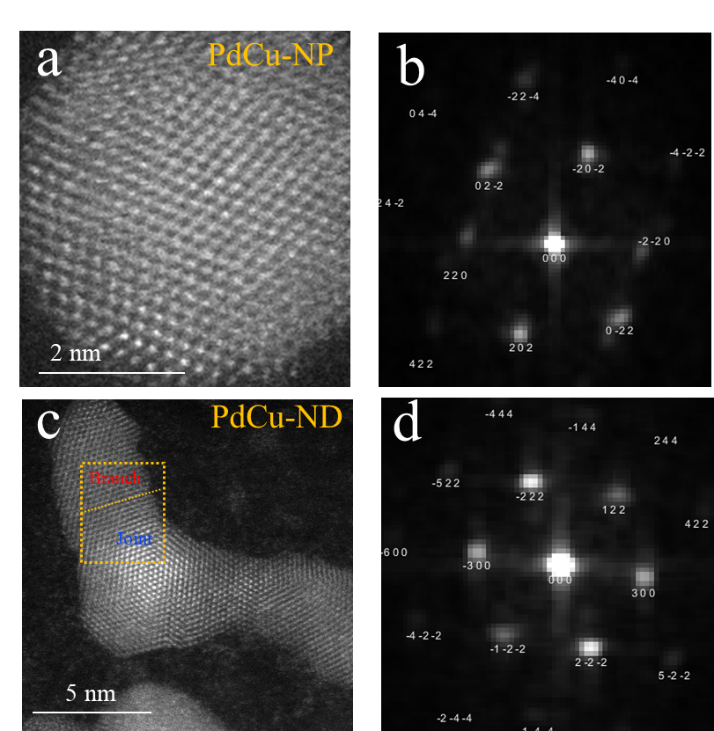


**Figure S5.** AC-HADDF-STEM images and corresponding of diffraction patterns of (**a, b**) PdCu-NP and (**c, d**) PdCu-ND.


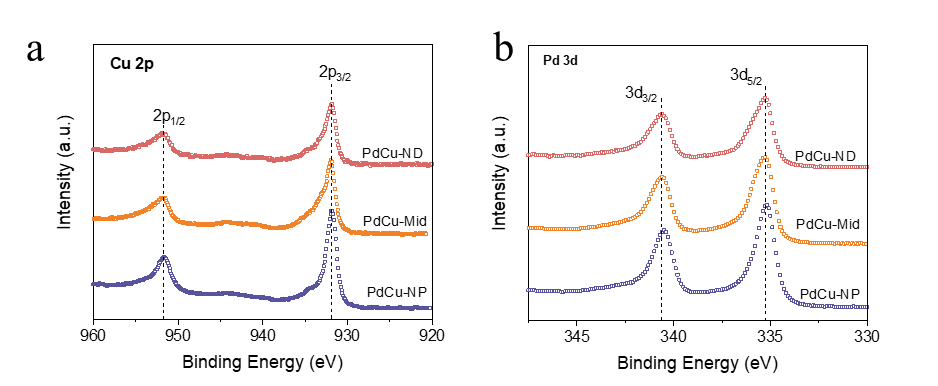


**Figure S6.** The XPS of (a) Cu 2p and (b) Pd 3d of PdCu-NP, PdCu-Mid and PdCu-ND


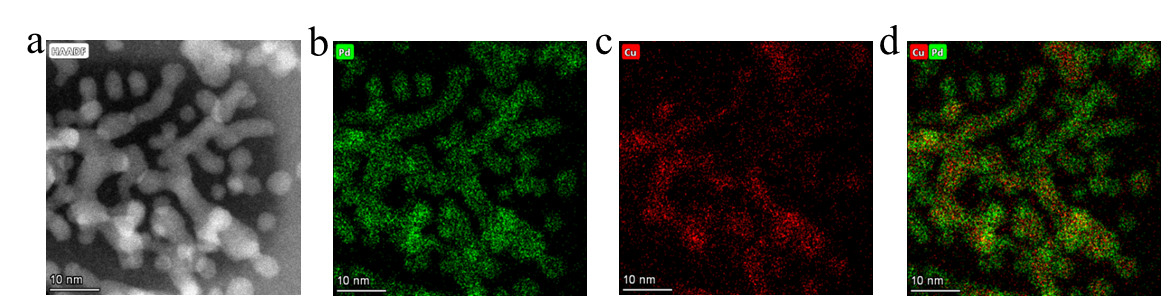


**Figure S7.** (**a**) HADDF-STEM images and (**b-d**) corresponding EDS mappings of PdCu-Mid.


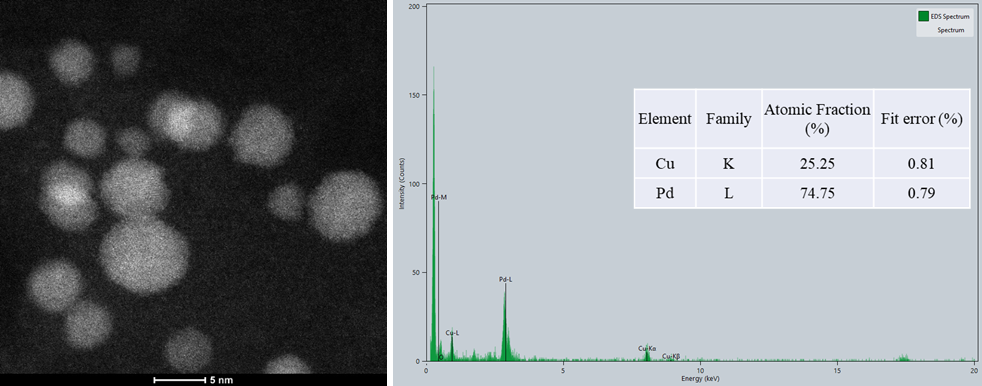


**Figure S8.** HR-HADDF-STEM and corresponding EDS scans and the overall atomic ratio of PdCu-NP (also denoted as Pd_3_Cu_1_-NP).

**
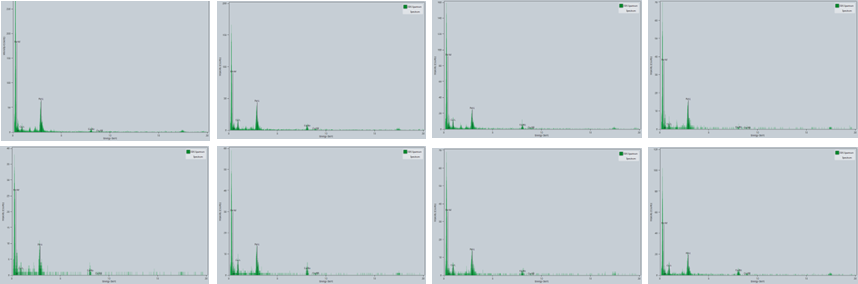
**

**Figure S9.** EDS spot scans of the marked nanoparticles of PdCu-NP.

**
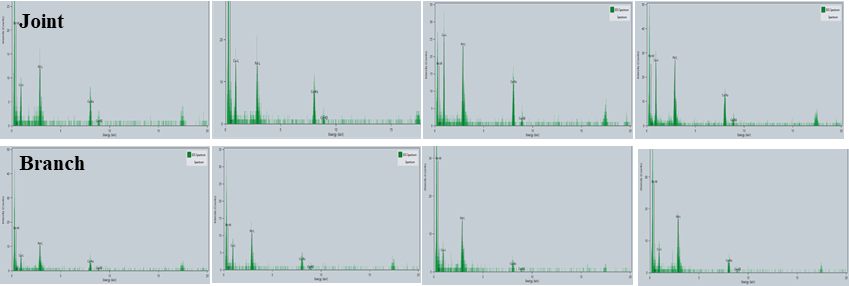
**

**Figure S10.** EDS spot scans of PdCu-ND on joint and branch parts.

**
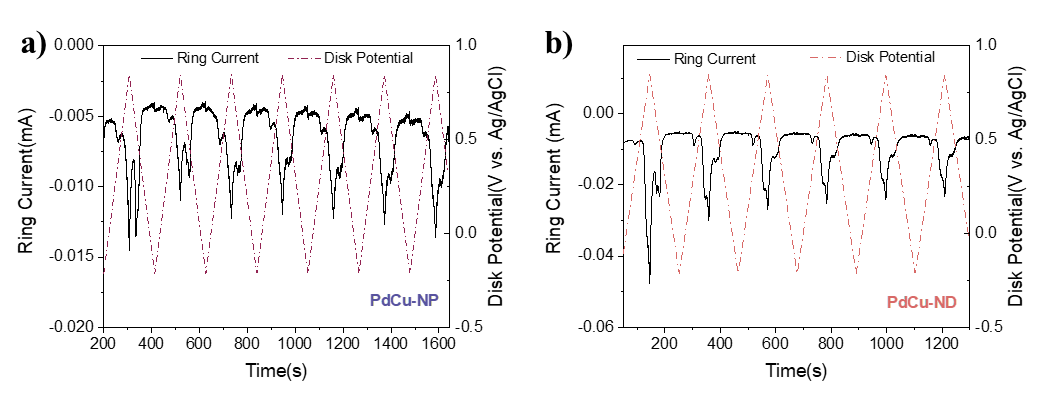
**

**Figure S11.** The stripping and collection model on ring-disk. Cu stripping and collection for (**a**) PdCu-NP and (**b**) PdCu-ND (note: a small peak before Cu(B) maybe caused by a trace of adsorbed Cu on PdCu alloys surface).


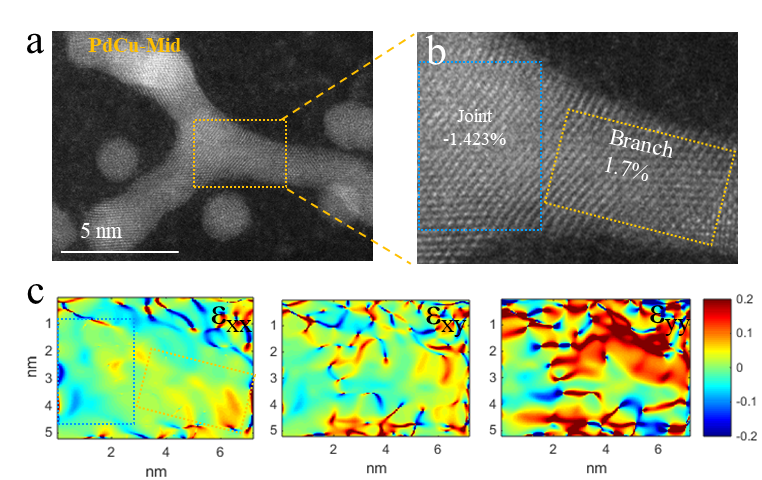


**Figure S12.** (**a, b**) Cs-HADDF-STEM image and (**c**) corresponding strain maps at the tensor of xx, xy and yy of PdCu-Mid respectively.


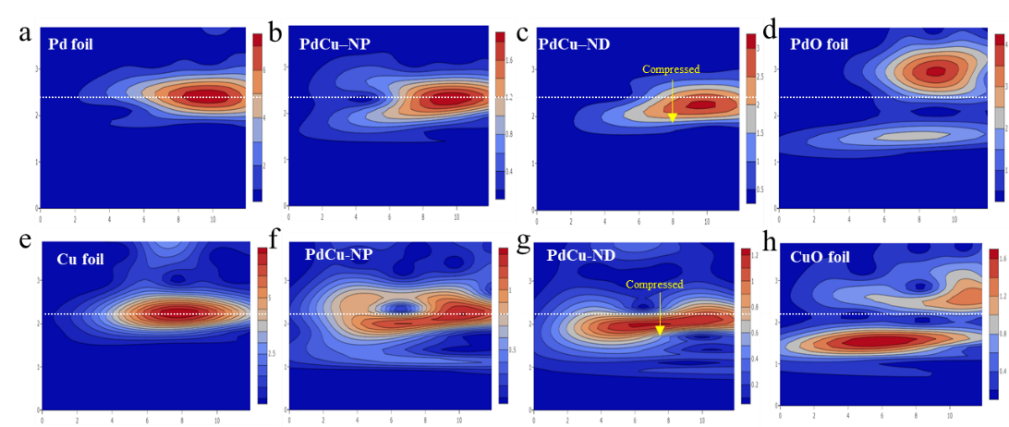


**Figure S13.** WT-EXAFS of PdCu-NP, PdCu-ND and corresponding foils at (**a-d**) Pd K-edge and (**e-h**) Cu K-edge (note: the horizontal coordinate is χ(k), and the vertical coordinate is χ(R)).

**Figure S14.** The LSV curves of CO_2_RR for PuCu-NP and PdCu-ND.

**
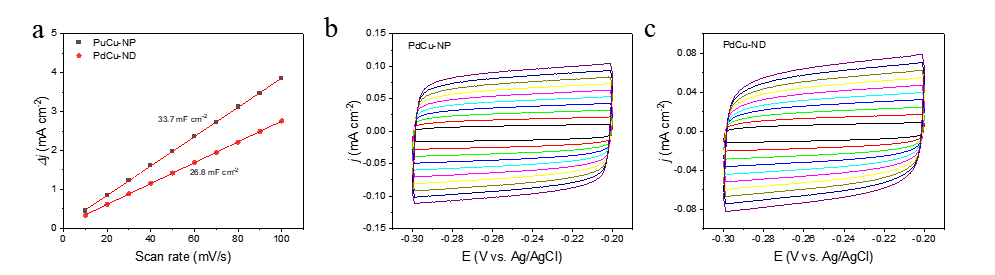
**

**Figure S15.** (**a**) The electrochemical double layer capacitances (C_dl_) for ECSA slopes and (**b, c**) corresponding CV plots of PdCu-NP amd PdCu-ND, respectively.


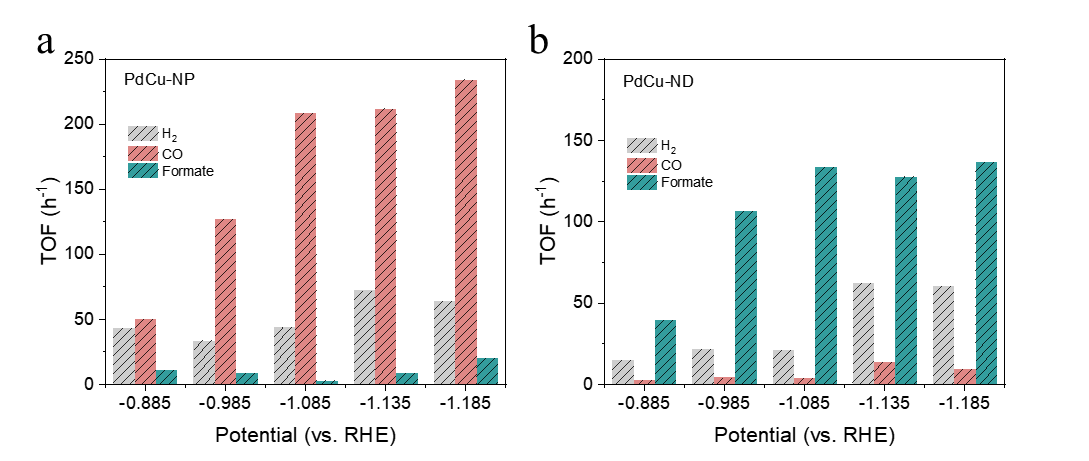


**Figure S16.** The TOF values of (**a**) PdCu-NP and (**b**) PdCu-ND.

**Figure S17.** The CO_2_RR performance of PdCu-Mid.


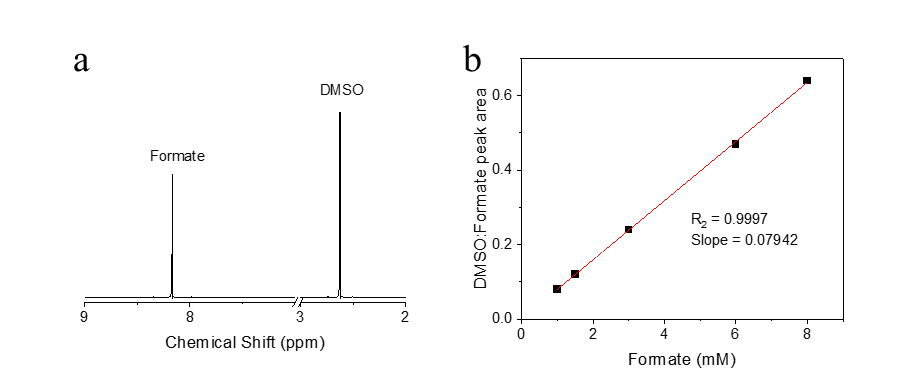


**Figure S18.** (**a**) H^1^ NMR spectrum of cathodic electrolyte after CO_2_RR. (**b**) The plot of concentration with the peak-area ratio for formate/DMSO.


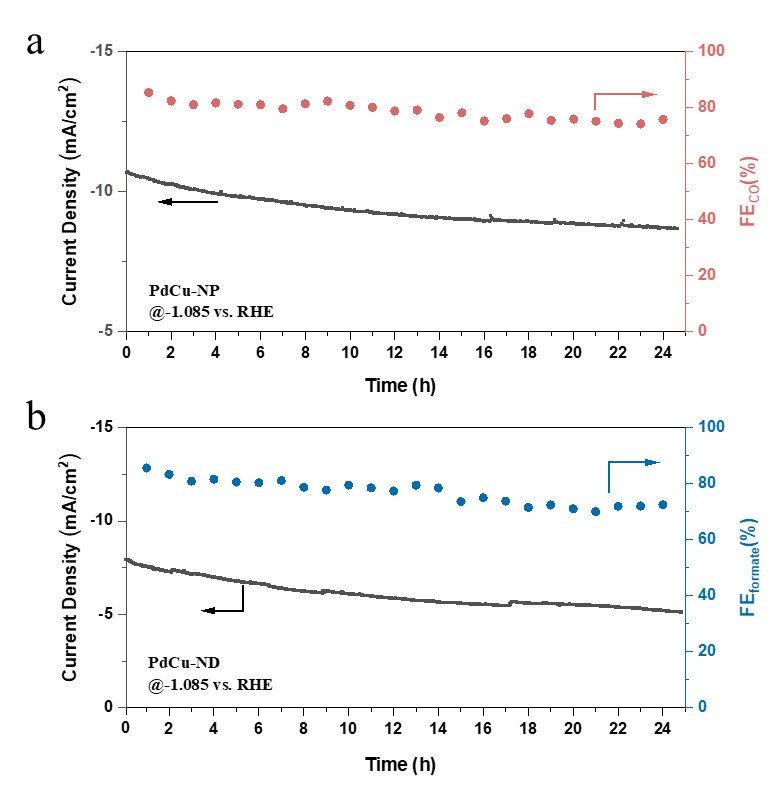


**Figure S19.** The current density and FE% of main CO_2_RR product of (**a**) PdCu-NP and (**b**) PdCu-ND for 24 hours reaction.

**
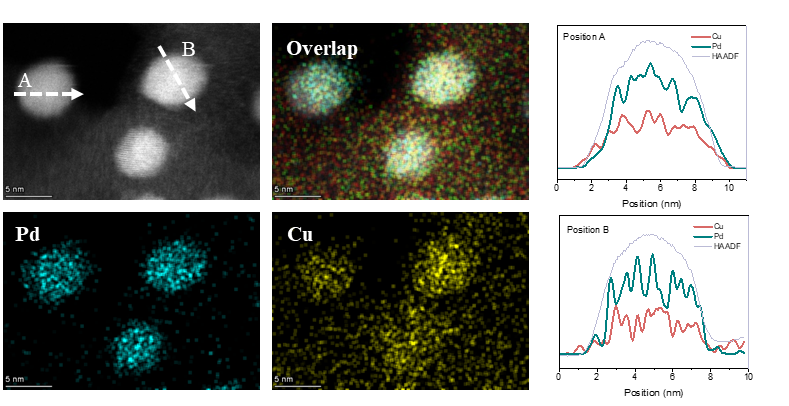
**

**Figure S20.** HAADF-STEM image and the corresponding EDS elemental mapping and STEM-EDS line-scanning of PdCu-NP after 24h reaction.


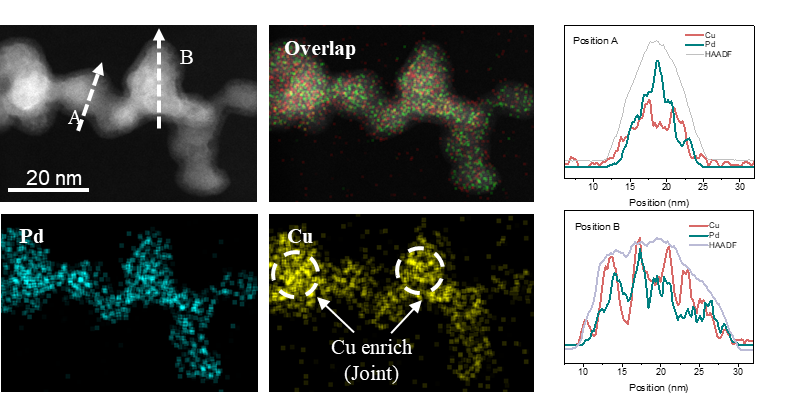


**Figure S21.** HAADF-STEM image and the corresponding EDS elemental mapping and STEM-EDS line-scanning of PdCu-ND after 24h reaction.


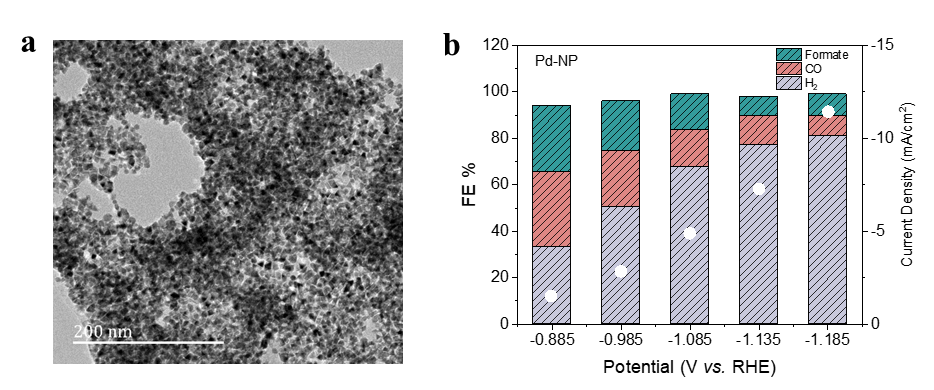


**Figure S22.** (**a**) TEM images of pure Pd-NP and (**b**) corresponding CO_2_RR performance.


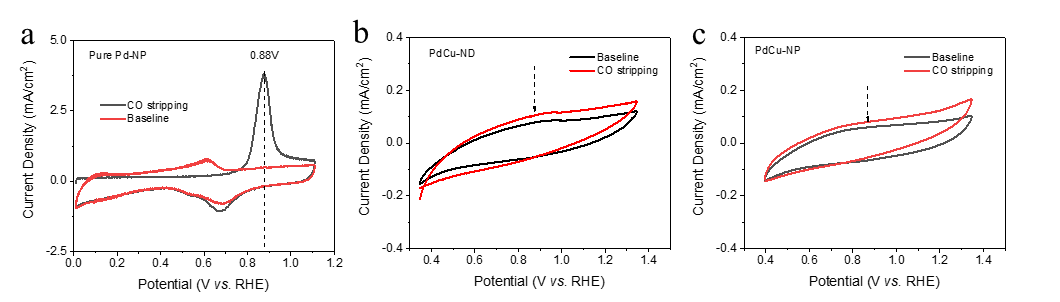


**Figure S23.** CO stripping of (**a**) pure Pd-NP and (**b**) PdCu-ND and (**c**) PdCu-NP.


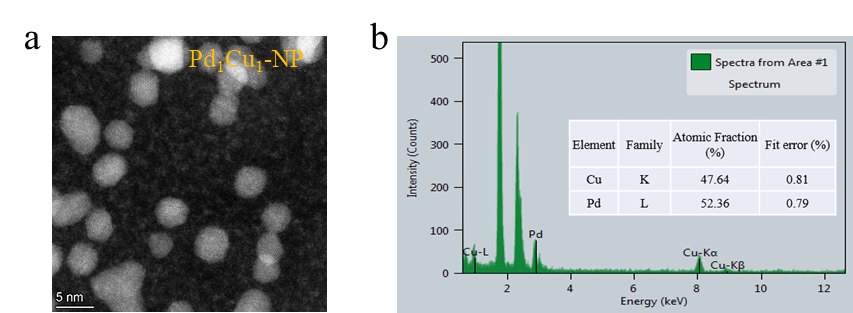


**Figure S24.** (**a**) Cs-HADDF-STEM and (**b**) corresponding EDS and the overall atomic ratio of Pd_1_Cu_1_-NP.


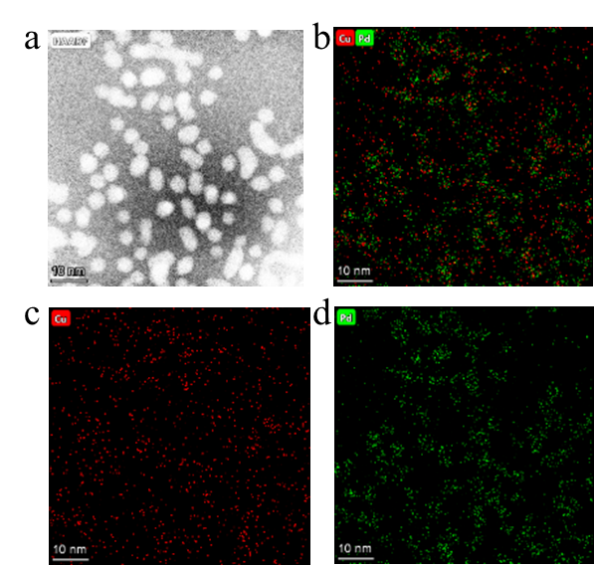


**Figure S25.** (**a**) HADDF-STEM and (**b-d)** corresponding EDS mappings of Pd_1_Cu_1_-NP.


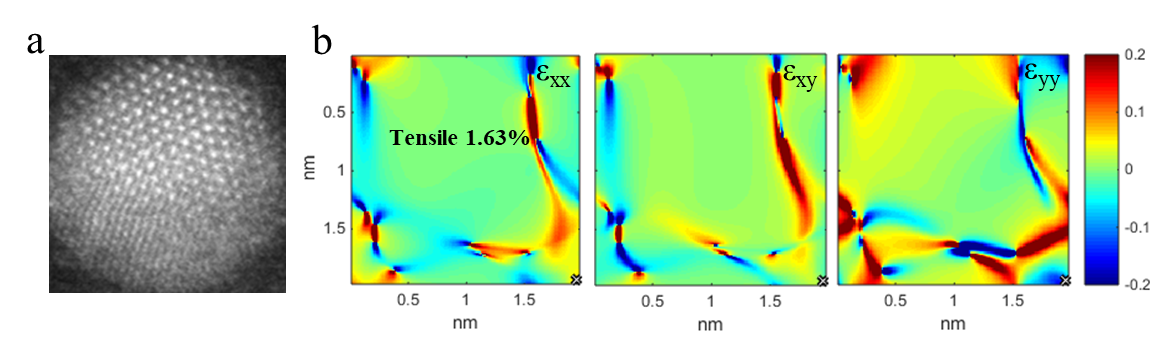


**Figure S26.** (**a**) AC-HADDF-STEM image and (**b**) corresponding strain maps at the tensor of xx, xy and yy of Pd_1_Cu_1_-NP respectively.

**Figure S27.** The CO_2_RR performance of Pd_1_Cu_1_-NP.

**
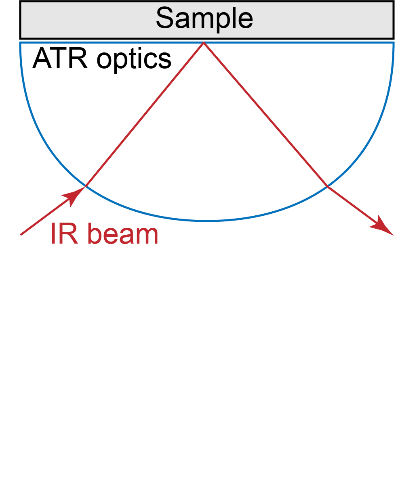
**

**Figure S28.** The diagram of ATR-SEIRAS.


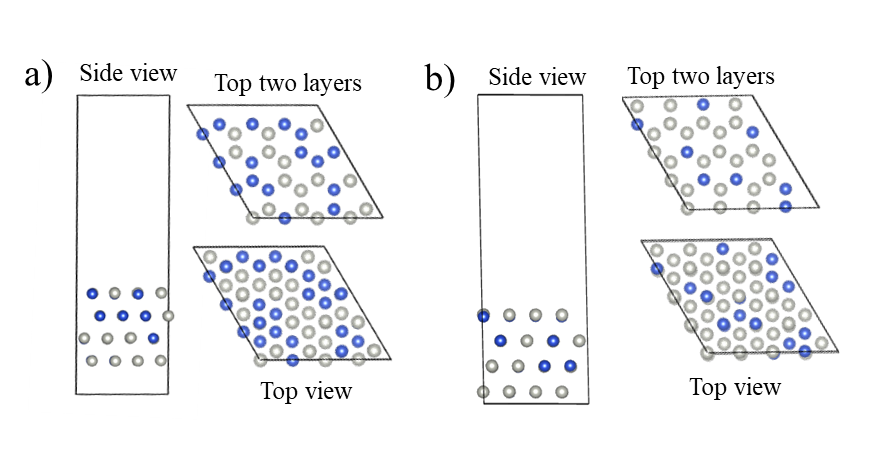


**Figure S29.** The optimized structures for corresponding (**a**) compressive Pd_1_Cu_1_ alloy and (**b**) tensile Pd_3_Cu_1_ alloy.

**Figure S30.** Free energy difference between *COOH and *OCHO under different strain (w/o structure optimization) of Pd_1_Cu_1_ and Pd_3_Cu_1._

**Figure S31.** The *d* band centers change along with different strain of Pd_3_Cu_1._

Supplementary Table

| Table S1. Reported PdCu alloys for CO_2_RR and corresponding products distribution. | | | |
| --- | --- | --- | --- |
| Catalysts | Pd/Cu atomic ratio | Major C-Product,  Faradaic efficiency | Ref. |
| Pd_85_Cu_15_/C nanoparticles[7] | 85/15 | CO, 86% (at -0.9 RHE) | Nano Energy 27 (2016) 35–43 |
| PdCu nanoparticles[8] | 85/15 | CO, ~85% (at -200mA/cm^2^) | Journal of Alloys and Compounds, 2022, 911,164990 |
| PdCu nanodendrites[9] | 82/18 | Formate, >90% (-0.1 to -0.7V RHE) | ACS Appl. Mater. Interfaces, 2022, 14, 8896−8905 |
| PdCu nanodendrites[10] | 70/30 | Formate, >90% (at -0.4V RHE) | Nano Lett. 2021, 21, 4092−4098 |
| flower-like Pd_3_Cu nanoparticles[11] | 3/1 | CO, ~80%; (at -1.1V RHE) | Small 2018, 14, 1703314 |
| Pd_3_Cu nanoparticles[12] | 3/1 | CO, ~90% (at -0.5V RHE) | J. Am. Chem. Soc. 2017, 139, 47−50 |

| Table S2. The ICOOP values of *COOH and *OCHO on different PdCu alloys. |
| --- |

|  | ICOOP(eV) | |
| --- | --- | --- |
|  | *COOH | *OCHO |
| Tensile Pd_3_Cu_1_ | 0.23368 | 0.18058 |
| Compressive Pd_1_Cu_1_ | 0.22675 | 0.19744 |

REFERENCES

1. Kresse G, Furthmiiller J. Efficiency of ab-initio total energy calculations for metals and semiconductors using a plane-wave basis set. *Comp Mater Sci* 1996; **6**: 15-50.

2. Kresse G, Joubert D. From ultrasoft pseudopotentials to the projector augmented-wave method. *Phys Rev B* 1999; **59**: 1578-1775.

3. Kresse G, Furthmuller J. Efficient iterative schemes for ab initio total-energy calculations using a plane-wave basis set. *Phys Rev B* 1996; **54**: 11169-11186.

4. Blochl PE. Projector augmented-wave method. *Phys Rev B* 1994; **50**: 17953-17979.

5. Perdew JP, Burke K, Ernzerhof M. Generalized Gradient Approximation Made Simple. *Phys Rev Lett* 1996; **77**: 3865-3868.

6. Monkhorst HJ, Pack JD. Special points for Brillouin-zone integrations. *Phys Rev B* 1976; **13**: 5188-5192.

7. Yin Z, Gao D, Yao S et al. Highly selective palladium-copper bimetallic electrocatalysts for the electrochemical reduction of CO_2_ to CO. *Nano Energy* 2016; **27**: 35-43.

8. Cho B, Lee J, Roh IP et al. A facile aqueous-phase synthesis method for small PdCu alloy nanocatalysts to enhance electrochemical CO_2_ reduction reactivity. *J Alloys Compd* 2022; **911**: 164990.

9. Sun Y, Wang F, Liu F et al. Accelerating Pd Electrocatalysis for CO_2_-to-Formate Conversion across a Wide Potential Window by Optimized Incorporation of Cu. *ACS Appl Mater* 2022; **14**: 8896-8905.

10. Zhou R, Fan X, Ke X et al. Two-Dimensional Palladium–Copper Alloy Nanodendrites for Highly Stable and Selective Electrochemical Formate Production. *Nano Lett* 2021; **21**: 4092-4098.

11. Zhu W, Zhang L, Yang P et al. Morphological and Compositional Design of Pd–Cu Bimetallic Nanocatalysts with Controllable Product Selectivity toward CO_2_ Electroreduction. *Small* 2018; **14**: 1703314.

12. Ma S, Sadakiyo M, Heima M et al. Electroreduction of Carbon Dioxide to Hydrocarbons Using Bimetallic Cu–Pd Catalysts with Different Mixing Patterns. *J Am Chem Soc* 2017; **139**: 47-50.
